# Salivary and plasma levels of matrix metalloproteinase-9 and myeloperoxidase at rest and after acute physical exercise in patients with coronary artery disease

**DOI:** 10.1371/journal.pone.0207166

**Published:** 2019-02-06

**Authors:** Zeid Mahmood, Helena Enocsson, Maria Bäck, Rosanna W. S. Chung, Anna K. Lundberg, Lena Jonasson

**Affiliations:** 1 Division of Cardiovascular Medicine, Department of Medical and Health Sciences, Faculty of Medicine and Health Sciences, Linköping University,Linköping, Sweden; 2 Division of Neuro and Inflammation Sciences, Department of Clinical and Experimental Medicine, Linköping University, Linköping, Sweden; 3 Department of Medical and Health Sciences, Division of Physiotherapy, Faculty of Health Sciences, Linköping University, Linköping, Sweden; 4 Department of Occupational Therapy and Physiotherapy, Sahlgrenska University Hospital, Gothenburg, Sweden; CVPath Institute Inc., University of Maryland, UNITED STATES

## Abstract

**Background:**

Low-grade systemic inflammation is a predictor of recurrent cardiac events in patients with coronary artery disease (CAD). Plasma proteins such as matrix metalloproteinase (MMP)-9 and myeloperoxidase (MPO) have been shown to reflect basal as well as stress-induced inflammation in CAD. Measurements of MMP-9 and MPO in saliva might pose several advantages. Therefore, we investigated whether salivary levels of MMP-9 and MPO corresponded to plasma levels in patients with coronary artery disease (CAD), both at rest and after acute physical exercise.

**Methods:**

A bicycle ergometer test was used as a model for stress-induced inflammation. Twenty-three CAD patients performed the test on two occasions 3–6 months apart. Whole unstimulated saliva was collected before, directly after and 30 min after exercise while plasma was collected before and after 30 min. MMP-9 and MPO in saliva and plasma were determined by Luminex.

**Results:**

MMP-9 and MPO levels were 2- to 4-fold higher in saliva than in plasma. Amongst the saliva samples, and also to a great extent amongst the plasma samples, the levels of both types of protein showed strong intercorrelations between the levels at rest and after exercise during the two visits. However, there were no (or weak) correlations between salivary and plasma MMP-9 and none between salivary and plasma MPO.

**Conclusion:**

We conclude that salivary diagnostics cannot be used to assess systemic levels of MMP-9 and MPO in CAD patients, neither at rest nor after acute physical exercise.

## Introduction

Inflammation is an important component of atherosclerosis, from the formation of atherosclerotic plaques in the arterial wall to plaque destabilization eventually leading to plaque rupture and atherothrombotic events, such as myocardial infarction [1]. The inflammatory activity is not only detected in the arterial wall but also in peripheral blood. The concentrations of inflammatory markers in serum or plasma can be useful to assess cardiovascular risk and monitor disease activity and over the years, great efforts have been made to identify relevant and easily available markers. The predictive value of markers like C-reactive protein (CRP) and interleukin (IL)-6 in determining the risk of myocardial infarction is well-documented [2]. Moreover, a number of epidemiological and clinical studies have shown that neutrophil-associated proteins in plasma, such as matrix metalloproteinase (MMP)-9 and myeloperoxidase (MPO), predict cardiovascular outcome [3, 4] and also relate to the extent and severity of atherosclerosis [5]. Overall, there is emerging evidence that subclinical elevations of inflammatory markers in peripheral blood should be considered in clinical praxis when assessing a patient´s risk of recurrent cardiovascular events.

Subclinical low-grade inflammation is however not always detectable in plasma measurements taken at rest. As it has been shown in various settings, stress provocation tests may add important information on the individual´s susceptibility to inflammatory response [6–9]. A greater inflammatory response to daily stressors has also been proposed to render atherosclerotic plaques unstable and more prone to rupture [10]. Measurements of MMP-9 and MPO may be useful in the assessment of stress-induced inflammatory response since they are stored in neutrophils and rapidly released upon stressful stimuli. E.g., neutrophils from patients with coronary artery disease (CAD) are more prone to release these mediators, particularly MMP-9, upon in vitro stimulation compared with neutrophils from healthy subjects [11]. Moreover, patients with CAD who exhibit a significant and early increase in plasma MMP-9 after a laboratory stress test show signs of more advanced diseases, including premature cellular aging and larger atherosclerotic burden [12].

As an alternative or complement to blood-based tests, salivary diagnostics has emerged as a promising tool in assessing inflammation. Compared with blood, saliva provides some distinct advantages such as non-invasiveness, ease and multiple sampling opportunities [13]. So far, much focus has been on saliva as a diagnostic tool for oral disease, including oral cancer [14] but growing evidence indicates that it can be useful also for systemic diseases [15–17]. Interestingly, a few studies have indicated that salivary levels of MMP-9 and MPO are potentially promising biomarkers in cardiovascular disease. When Floriano et al [18] used a saliva-based nano-biochip test for the diagnosis of acute myocardial infarction, they found that both MMP-9 and MPO were significantly elevated in saliva collected within 48 h of chest pain onset. Another study demonstrated a significant association between salivary levels of MMP-9 and subclinical cardiovascular diseases, more precisely carotid intima-media thickness, in 250 individuals [19]. Still, the field of salivary biomarkers and cardiovascular disease is young and results are not fully conclusive, as pointed out in a recent systematic review by [20]. An important question that remains to be answered is whether the salivary levels of MMP-9 and MPO reflect the levels in peripheral blood.

The aim of the present study was to investigate whether salivary levels of MMP-9 and MPO corresponded to plasma levels in patients with CAD. We measured the concentrations in saliva and plasma, under basal conditions, and following exposure to a single acute session of exercise, on two separate occasions 3–6 months apart.

## Methods

### Study population

Twenty-three patients with a recent coronary event, i.e. acute coronary syndrome and/or revascularization with either percutaneous coronary intervention or coronary artery bypass grafting, were consecutively recruited from the Outpatients´ Cardiology Clinic at the University Hospital in Linköping, Sweden. Exclusion criteria were age > 75years, severe heart failure, neoplastic disease, major clinical depression, chronic liver and renal failure, chronic immunologic disorders or treatment with immunosuppressive/anti-inflammatory agents, serious physical or psychological diseases interfering with performing an exercise test, and inability to understand the Swedish language. The study was conducted in accordance with the ethical guidelines of Declaration of Helsinki, and the research protocols were approved by the Ethical Review Board in Linköping. Written informed consent was obtained from all patients and controls.

### Exercise stress test

The patients underwent exercise stress tests on two occasions; 3–4 weeks (visit A) and 4–6 months (visit B) after the index coronary event. A standardized submaximal cycle ergometer test was used according to the WHO protocol [21], with an increased workload of 25 W every 4.5 min. The initial starting load, 25 W or 50 W, was decided based on the patient’s exertion history. A pedalling rate of 50 rates per minute was used. After 2 and 4 minutes at each work load, heart rate, rating of perceived exertion according to Borg’s rating of perceived exertion scale (RPE) [22] and subjective symptoms, including chest pain and dyspnea according to Borg’s Category Ratio Scale, CR-10, scale were rated. After 3 minutes, the systolic blood pressure was registered. The exercise test was discontinued at Borg RPE 17 and/or dyspnea 7 on Borg’s CR-10 scale. This submaximal exercise test on a bicycle ergometer has excellent test-retest reliability in patients with CAD [23] and is a well-established, safe procedure that is frequently used in exercise-based cardiac rehabilitation in Sweden.

### Sampling procedure

The patients first lay down on a bed reclining comfortably for 20–30 min. Thereafter, saliva samples were collected prior to the bicycle test, directly after, and 30 min after the completion of the test whereas blood samples were collected prior to and 30 min after the test. Saliva samples for basal measurements were always taken before initiating venepuncture to avoid a possible stress effect. Patients had been asked to avoid tooth brushing, smoking, eating and drinking for at least 2 h before sample collection. The saliva was collected with Salimetrics Oral cotton Swabs (Salimetrics via Electrabox, Stockholm, Sweden) placed under the tongue for 2 minutes. The saliva samples were then immediately placed on ice. Whole blood was collected in 9 mL EDTA tubes (BD biosciences) and plasma was obtained after centrifugation for 10 min at 1500 x g. Saliva and plasma samples were frozen at -70°C until analysis.

### Measurement of MMP-9 and MPO in plasma

MMP-9 and MPO in plasma was measured by magnetic bead-based luminex assay (R&D Systems, Minneapolis, USA) following the manufacturer’s instructions. All samples were assayed in a dilution of 1:50. The interassay coefficients of variation (CV) were 13.8% for MMP-9 and 15% for MPO. The limits of detection were 0.136 ng/mL for MMP-9 and 0.148 ng/mL for MPO.

Since plasma volume (PV) loss can be a confounding variable in the interpretation of changes in plasma concentration, the values after exercise were corrected for PV loss. Hematocrit values before and after exercise were assayed from whole blood using a standard cell counter. PV values were defined as (1 –hematocrit) and thereafter percent change in PV or delta PV from rest to exercise was calculated. To adjust for PV loss, MMP-9 and MPO values after exercise were multiplied by (1+ (delta PV/100)) [24].

### Measurement of MMP-9 and MPO in saliva

MMP-9 and MPO in saliva were measured by the same kit as described above, i.e. magnetic bead-based luminex assay (R&D Systems). Briefly, saliva samples were gently thawed in 4°C and thereafter centrifuged 10 000 x g for 5 minutes. All samples were assayed in a dilution of 1:50 and were re-analyzed in a dilution of 1:100 or 1:10 if the assay values were higher or lower than the range of standard curve respectively. For all patients except one, the basal and post-exercise saliva were assayed on the same plate. To correct for differences in saliva volume (e.g. due to dry mouth after the exercise test), the concentrations of MMP-9 and MPO were normalized to total protein content in saliva using the Bradford protein assay (Bio-Rad, Solna, Sweden). All samples were analyzed in duplicates, and diluted 1:5 or 1:3 prior to the Bradford assay. If sample values were outside the range of the standard curve, they were re-analyzed in 1:10 dilution or undiluted. The interassay CV was 8.5% for MMP-9 and 7.2% for MPO.

### Statistics

SPSS 25 for Windows (IBM Corp. Armonk, NY) was used for statistical analyses. For clinical and laboratory characteristics, data are presented as median (interquartile range). The statistical significance of any difference between two groups was tested by using Mann-Whitney U-test. Chi-square test was used for nominal data. Wilcoxon signed-ranks test was used for pair-wise comparisons and Friedman test for more than two correlated samples. Spearman's rank correlation coefficient was used for correlation analyses. A p-value < 0.05 was considered statistically significant.

## Results

### Basal characteristics and exercise testing

The basal characteristics of the 23 patients, including cardiovascular medication and laboratory data collected during visit A, are summarized in Table 1 (S1 Dataset). The results of the exercise stress tests at visit A and B are shown in Table 2 (S1 Dataset). Among the 23 patients who participated in visit A, 19 of them participated in visit B while 4 declined to participate further. On both occasions, the heart rates and systolic blood pressures increased significantly during cycling. At 30 min after exercise, the systolic blood pressures returned to basal levels while the heart rates were still significantly higher compared with the basal heart rates. The durations of exercise as well as the maximum watt levels were similar at visit A and B.

**Table 1:** The basal characteristics of 23 CAD patients.

Age, years	65 (60–69)
Males, n (%)	18 (75)
Smokers, n (%)	5 (21)
Body mass index, kg/m^2^	25.9 (23.4–27.9)
*Index event*	
Acute coronary syndrome, n (%)	15 (65)
Stable angina, n (%)	8 (35)
*Medication*	
Dual anti-platelet therapy, n (%)	22 (92)
Beta-blocker, n (%)	18 (75)
ACE-I/ARB, n (%)	18 (75)
Calcium-channel blocker, n (%)	1 (4)
Statins, n (%)	24 (100)
*Laboratory data*	
Total cholesterol, mmol/L	3.5 (3.1–4.3)
C-reactive protein, mg/L	1.0 (0.4–1.7)

Data include demographic and clinical data, cardiovascular medication and laboratory data at visit A. Values are given as median (interquartile range) or n (%). Acute coronary syndrome; non-ST-elevation myocardial infarction or ST-elevation myocardial infarction. Dual anti-platelet therapy; low-dose aspirin + clopidogrel or ticagrelor, ACE-I/ARB; angiotensin converting enzyme inhibitors/angiotensin receptor blockers.

**Table 2:** The results of exercise stress tests at visit A and B.

		Visit A (n = 23)	Visit B (n = 19)
Heart rate, beats/min	Basal	63 (55–72)	64(55–77)
	Peak	121 (111–131)***	125(110–144)***
	30 min recovery	69 (60–83)**	74(64–94)**
Systolic blood pressure, mm Hg	Basal	130 (115–140)	128(119–141)
	Peak	193 (175–203)***	183(174–206)***
	30 min recovery	120 (115–130)	125(112–140)
Duration of exercise, min		15 (12–18)	15 (12–19)
Maximum watts achieved		125 (100–125)	125(100–150)

Values are given as median (interquartile range).

*** p < 0.001 compared to basal levels

** p < 0.01 compared to basal levels.

### Salivary levels of MMP-9 and MPO

**Fig 1:**
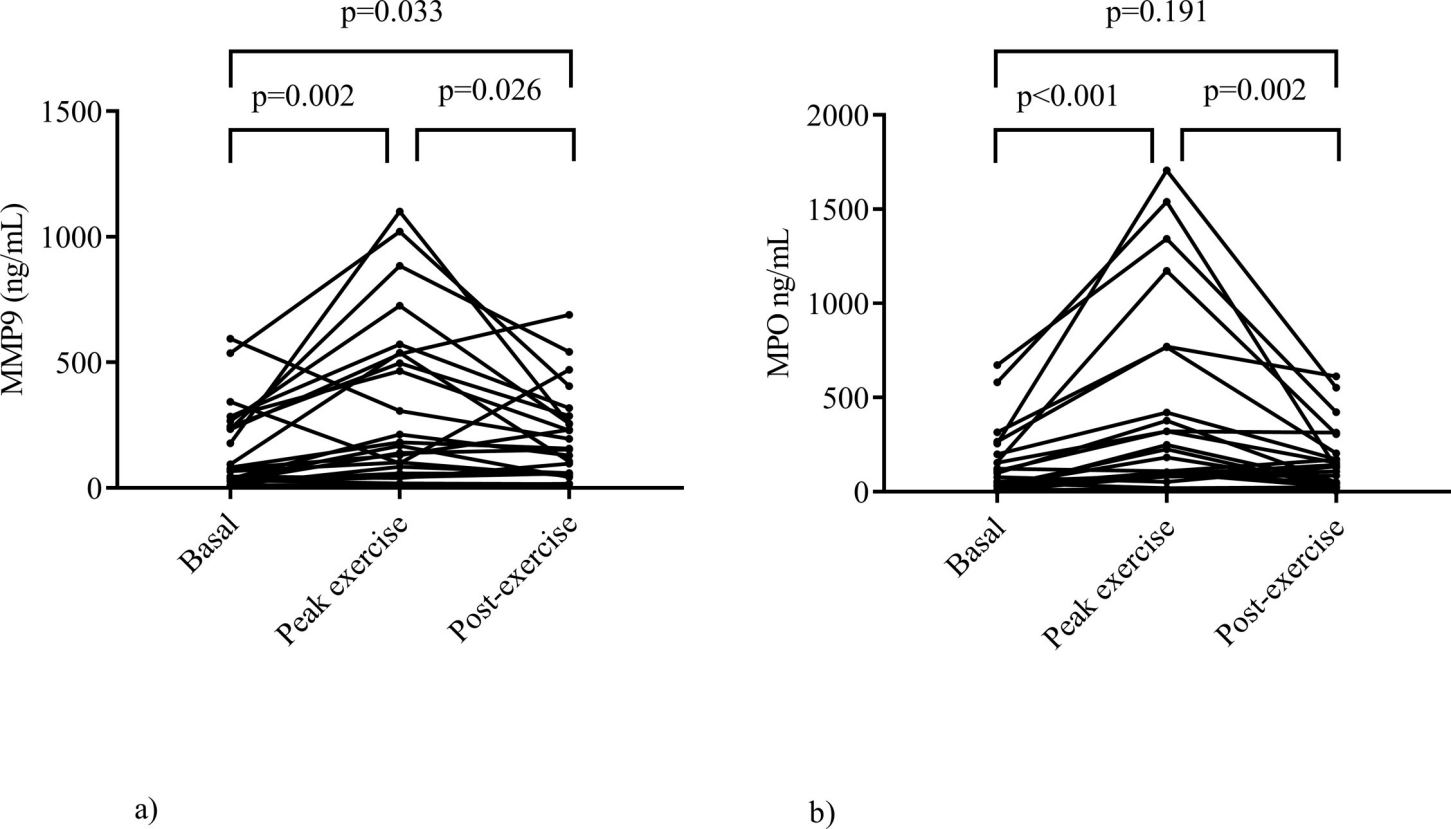
(a and b) MMP-9 and MPO in saliva before and after an exercise stress test. MMP-9 and MPO in saliva are presented in Fig 1A and 1B, i.e. without correction for total protein content, before (basal), directly after exercise (peak exercise) and 30 min after exercise (post-exercise). Each dot represents one patient.

The total protein content in saliva and protein-adjusted salivary levels of MMP-9 and MPO before (i.e. basal) and after exercise stress tests on two test occasions (visit A and B) are presented in Table 3 (S1 Dataset). The total protein levels in saliva increased significantly directly after exercise and returned to basal levels 30 min after exercise, probably reflecting a reduced salivary flow during exercise. The protein-adjusted levels of MMP-9 and MPO in saliva did not differ significantly between at rest and after exercise, neither did levels differ between visit A and B. However, without adjustment for total protein content, the salivary levels of MMP-9 and MPO showed significant increases after exercise as shown in Fig 1A and 1B (S1 Dataset). In the following text, the salivary levels of MMP-9 and MPO will be referred to as protein-adjusted levels.

**Table 3:** Measurement in saliva and plasma before and after an exercise stress test.

			Visit A	Visit B	p^a^
**Total protein**	Saliva *𝜇g/mL*	Basal	754 (410–1421)	728 (573–1365)	0.586
		Peak exercise	1508 (946–2325)	1284 (890–1785)	0.286
		Post-exercise	852 (598–1220)	881 (653–1168)	0.872
		p^b^	<0.001	<0.001	
**MMP-9**	Saliva ng/mg total protein	Basal	92 (53–211)	104 (51–295)	0.492
		Peak exercise	103 (52–229)	104 (42–342)	0.647
		Post-exercise	141 (84–279)	117 (82–271)	0.936
		p^b^	0.260	0.327	
	Plasma *ng/mL*	Basal	50 (37–82)	43 (28–84)	0.601
		Post-exercise	50 (25–73)	51 (22–91)	0.334
		p^c^	0.287	0.904	
**MPO**	Saliva ng/mg total protein	Basal	82 (31–222)	124 (32–378)	0.084
		Peak exercise	118 (47–317)	99 (422–375)	0.557
		Post-exercise	109 (42–203)	107 (43–256)	0.573
		p^b^	0.084	0.662	
	Plasma *ng/mL*	Basal	27 (22–36)	23 (17–29)	0.036
		Post-exercise	27 (21–35)	24 (18–28)	0.227
		p^c^	0.638	0.673	

Salivary levels of total saliva and protein-adjusted levels of MMP-9 and MPO were measured before (basal), directly after exercise (peak exercise) and 30 min after exercise (post-exercise). Plasma levels of MMP-9 and MPO were measured before and 30 min after exercise, the latter adjusted for plasma loss. Exercise tests were performed on two occasions (visit A and B). Values are given as median (inter-quartile range).

^a^ Wilcoxon signed-ranks test were used for comparisons between visit A and B

^b^ Friedman´s test for comparisons between basal, peak exercise and post-exercise levels

^c^ Wilcoxon signed-ranks test for comparisons between basal and post-exercise levels in plasma samples.

### Plasma levels of MMP-9 and MPO

The levels of MMP-9 and MPO were in general lower in plasma than in saliva by around two-fold and four-fold, respectively. The plasma levels of MMP-9 and MPO before and after acute exercise are presented in Table 3 (S1 Dataset). There was no significant difference, neither between the levels at rest and after exercise, nor between visit A and B. The levels after exercise were corrected for PV loss, although the delta PV values were relatively low on both test occasions, visit A -1.6 ((-1.8)-1.7) % and visit B -1.7 (-3.3–0.00) %.

### Correlations between MMP-9 and MPO in saliva and plasma

The correlations between MMP-9 and MPO in saliva and plasma before and after exercise on the two test occasions are presented in Tables 4 and 5, respectively (S1 Dataset). The salivary levels of MMP-9 before and after exercise were highly intercorrelated between the two occasions. The plasma levels of MMP-9 before and after exercise were also intercorrelated between the two occasions but to a lesser extent. However, only a number of weak correlations were found when plasma and salivary levels of MMP-9 were compared (Table 4). The same pattern was also observed for MPO, i.e., the levels of MPO before and after exercise were highly correlated in plasma and saliva per se, but no correlation was seen between salivary and plasma levels (Table 5). Furthermore, the levels of MMP-9 and MPO were correlated in saliva (basal *r* = 0.707, peak *r* = 0.693, and post-exercise *r* = 0.750, respectively, all p < 0.001), as well as in plasma collected in visit A (basal, *r* = 0.461, p = 0.027 and post-exercise, *r* = 0.593, p = 0.003, respectively). Similar correlations between MMP-9 and MPO were seen for visit B in both saliva (basal, *r =* 0.674, p = 0.003; post-exercise, *r =* 0.391, p = 0,098) and plasma (basal, *r =* 0.691, p = 0.001; post-exercise, *r =* 0.623, p = 0.004).

**Table 4:** Correlations of MMP-9 levels in saliva and plasma.

	Saliva A basal	Saliva A exercise	Saliva B basal	Saliva B exercise	Plasma A basal	Plasma A exercise	Plasma B basal	Plasma B exercise
**Saliva** A basal	1	0.751***	0.807***	0.719**	0.157	0.286	0.332	0.088
**Saliva A** exercise		1	0.899***	0.908***	0.252	0.416	0.414	0.243
**Saliva B** basal			1	0.847***	0.077	0.497*	0.584*	0.377
**Saliva B** exercise				1	0.202	0.434	0.533*	0.317
**Plasma** A basal					1	0.380	0.368	0.620**
**Plasma** A exercise						1	0.633**	0.624**
**Plasma** B basal							1	0.785***
**Plasma B** exercise								1

The table shows correlations (Spearman´s rho) between MMP-9 in saliva (adjusted for total protein) and plasma before (basal) and 30 min after an exercise stress test (exercise), on two test occasions (A and B).

* p < 0.05

** p < 0.01

*** p < 0.01.

**Table 5:** Correlations of MPO levels in saliva and plasma.

	Saliva A basal	Saliva A exercise	Saliva B basal	Saliva B exercise	Plasma A basal	Plasma A exercise	Plasma B basal	Plasma B exercise
**Saliva** A basal	1	0.685***	0.841***	0.807***	0.010	0.102	0.297	0.226
**Saliva A** exercise		1	0.605**	0.635**	0.260	0.352	0.376	0.346
**Saliva B** basal			1	0.836***	0.061	0.221	0.432	0.169
**Saliva B** exercise				1	0.147	0.294	0.383	0.463
**Plasma** A basal					1	0.945***	0.767***	0.841***
**Plasma** A exercise						1	0.886***	0.856***
**Plasma** B basal							1	0.895***
**Plasma B** exercise								1

The table shows correlations (Spearman´s rho) between MPO in saliva (adjusted for total protein) and plasma before (basal) and 30 min after an exercise stress test (exercise), on two test occasions (A and B).

** p < 0.01

*** p < 0.01.

In line with previous results from our group (12), the stress-induced release of MMP-9 in plasma showed a large variation among patients with CAD. We therefore divided the patients into two groups depending on whether MMP-9 in plasma increased or decreased after acute exercise. At visit A, 7 out of 23 patients (30%) exhibited a median of 70 (16–106) % increase in plasma MMP-9 after exercise, while the remaining 16 patients showed a median of 31 (15–45) % decrease. This was however not reflected in the saliva. The two groups showed similar and non-significant changes in salivary MMP-9 after exercise, a median of 31 ((-19)-55) % and 47 ((-26)-92) %, respectively, without any correlation to changes in plasma.

## Discussion

During the last decades, substantial improvements have been achieved in medical as well as lifestyle management of CAD, all of which may lead to reductions in inflammatory activity. Despite this, risk for recurrent cardiac events exists in many patients with CAD due to residual inflammation [25]. Thus, there is an urgent need to identify the population at risk. However, inflammatory markers in plasma and serum samples that are collected during resting conditions do not always provide the full picture. The inflammatory state may not become evident until the patient is exposed to stress. Both psychosocial and exercise-related stressors can be used to elicit an inflammatory response. Comparisons between CAD patients and healthy controls have shown that exposure to any of these stressor types results in larger CRP increases in patients than in controls [6, 7]. However, there are some methodological concerns in using CRP to examine inflammatory response to stress. Due to the response time of hepatic CRP release, it may take between 6 and 24 h before any changes in CRP appear in plasma. In contrast, neutrophil-associated proteins like MMP-9 and MPO are rapidly released in response to stress. Recently, we were able to show that a significant increase in MMP-9 30 min after a psychological stress test occurred in one third of patients with CAD [12]. Similarly, 30% of the patients in the present study showed a significant increase in MMP-9 30 min after an exercise stress test. So far, we can only speculate that the stress-induced increase in MMP-9 is a marker of residual inflammatory risk. However, the association between stress-induced release of MMP-9 and shorter leukocyte telomere length as well as increased plaque burden support the hypothesis that recurring bouts of inflammation in response to exogenous challenges contribute to a gradual progression in CAD [12].

Saliva has emerged as a non-invasive tool for assessing MMP-9 and MPO in cardiovascular diseases [18, 19, 26]. If salivary levels reflect systemic levels, this might open up for a new and simple way to monitor inflammation, in particular stress-induced inflammation, in patients with CAD. Therefore, the main aim of this study was to investigate the relationships between salivary and plasma concentrations of MMP-9 and MPO in patients with CAD. This was performed on two separate occasions 3–6 months apart, at rest as well as after an exercise stress test. As regards salivary protein levels, the levels obtained from all 4 sampling times (i.e. before and after exercise on both occasions) were intercorrelated with high significance for each protein (MMP-9 or MPO). The proteins were also intercorrelated in plasma. However, only a few weak correlations were found between levels obtained from saliva and plasma. This argues for a distinct oral compartment in which MMP-9 and MPO reside independently from blood. Only a few previous studies have compared salivary and systemic levels of MMP-9 and MPO, though with disparate conclusions. Foley *et al* [26] performed serial measurements of MMP-9 and MPO in serum and unstimulated whole saliva after alcohol septal ablation in 21 patients with obstructive hypertrophic cardiomyopathy. They reported significant correlations between serum and salivary levels of MMP-9 and MPO at all time points. Another study by Meschiari *et al* [27], including 23 patients with periodontal disease and 19 healthy controls, found significant correlations between plasma levels of MMP-9 and gelatinolytic activity in saliva samples collected by expectoration after chewing on paraffin (i.e. stimulated whole saliva) [27]. Two possible reasons for the discrepancy between the findings in our study and the previous studies deserve particular mentions. Firstly, serum levels of neutrophil proteins reported by Meschiari *et al* [27] may not be representative of systemic levels due to the degranulation of neutrophils and platelets during the ex vivo blood clotting process in the collection tubes. Secondly, none of the previous studies presented protein-adjusted salivary levels. The salivary water content can vary in response to stress stimulation and alteration of saliva flow. In the present study, the higher total salivary protein concentrations could be an indicator for water loss directly after exercise. If we did not adjust for total protein, levels of salivary MMP-9 and MPO would be misleadingly high.

The high correlations between salivary levels of MMP-9 and MPO on two different test occasions suggest that salivary levels are rather stable over time. It has been pointed out that the day-to-day fluctuation of inflammatory markers in saliva may be substantial due to intraindividual variability. However, the variation is greater with at-home collection compared with collection in the clinic under supervision [28]. In a previous study, Fingleton *et al* [29] reported that the within-subject variability in salivary MMP-9 activity was relatively small when 5 samples were collected over a 3-week period.

The present study is the first to measure the degree to which salivary MMP-9 and MPO levels correspond to plasma levels in response to exercise-induced stress. However, a number of smaller studies have investigated the reliability of saliva-based to blood-based levels of cytokines, like IL-1 beta and IL-6, in response to acute stress (either social-cognitive or exercise-physical). As summarized in a review by Slavish *et al* [30], measurements of salivary cytokines do not appear to mirror systemic cytokine levels. Salivary and plasma levels of cytokines correlate poorly with each other before as well as after stress. One probable explanation is that cytokines are too large to enter saliva via passive diffusion. The relatively higher molecular weights of MMP-9 and MPO, 82 and 140 kDa, respectively, compared to cytokines may thus partly explain why the salivary levels do not correlate with the corresponding plasma levels.

Our study has a number of potential limitations that need to be considered. Firstly, we did not control for oral health status. Periodontitis is known to be overrepresented among patients with CAD [31]. Both MMP-9 and MPO are abundantly present in periodontal tissue and salivary levels have been shown to correlate with periodontal inflammation [27, 32]. Still, there is some controversy over whether salivary MMP-9 levels are higher in patients with periodontitis. In a recent study of patients with and without periodontitis, no significant differences between the groups were found in either serum or salivary levels of MMP-9 [33]. Another limitation of our study is that we were unable to evaluate the impact of potential confounders, such as smoking, due to small sample size. The lack of a healthy control group may also be considered a limitation and, hence, our results may only be applied to patients with CAD. However, several factors, such as medications, might have complicated the interpretation of differences between patients and controls. Our CAD patients took a number of different medications, like statins and platelet inhibitors, that have anti-inflammatory effects [34, 35].

## Conclusion

MMP-9 and MPO were abundantly present in saliva of CAD patients with concentrations exceeding those in plasma. However, there was no evidence that salivary levels of MMP-9 and MPO reflect their circulating levels, neither at rest nor after exercise stress tests. As regards MMP-9 and MPO, salivary protein levels should rather be seen as a separate compartment and not as a mirror of systemic protein levels. Salivary diagnostics does not seem to be useful alternatives to plasma diagnostics in the assessment of systemic levels of MMP-9 and MPO in CAD patients.

## Supporting information

S1 Dataset SPSS Statistics Data file. An SPSS file with data used in the statistical analysis is included as a supplemental item.
(SAV)
